# Synergistic Effect of Laccase and Sugar Beet Pectin on the Properties of Concentrated Protein Emulsions and Its Application in Concentrated Coconut Milk

**DOI:** 10.3390/molecules23102591

**Published:** 2018-10-10

**Authors:** Pusen Chen, Wenxue Chen, Shan Jiang, Qiuping Zhong, Haiming Chen, Weijun Chen

**Affiliations:** College of Food Science and Technology, Hainan University, Haikou 570228, China; chenpusen22@163.com (P.C.); hnchwx@163.com (W.C.); 15848911030@163.com (S.J.); hainufood88@163.com (Q.Z.)

**Keywords:** concentrated coconut milk, model emulsions, protein, sugar beet pectin, laccase

## Abstract

Concentrated coconut milk (CCM), a raw material from coconut products, is extremely unstable because of its high oil content (>30%). In this study, three model emulsions—primary emulsions stabilized by coconut proteins only, secondary emulsions stabilized by the conjugation of sugar beet pectin (SBP) and coconut protein, and laccase-treated secondary emulsions—were prepared to investigate the effects of different factors (coconut proteins, coconut proteins + SBP, laccase-treated emulsions) on the stability of model emulsions and the application of this method to real CCM. The stability of the emulsions was evaluated based on their interfacial tension, zeta potential, particle size distribution, rheological properties, and the assembly formation of SBP and coconut protein at the oil–water interface. Results showed that addition of SBP or laccase can increase the viscosity and reduce the interfacial tension of the emulsion, and the effect was concentration dependent. Zeta potential of the emulsion decreased with the increase of protein (from −16 to −32 mV) and addition of SBP (from −32 to −46 mV), and it was reduced when laccase was added (from −9.5 to −6.0 mV). The secondary emulsion exhibited the narrowest particle size distribution (from 0.1 to 20 μm); however, laccase-catalyzed secondary emulsions showed the best storage stability and no layering when the laccase content reached 10 U/100 g. Confocal laser scanning microscopy (CLSM) revealed that protein was adsorbed on the oil–water interface and SBP distributed in the continuous phase could undergo oxidative crosslinking by laccase. These results show that the stability of the concentrated emulsion can be effectively improved by adding SBP and laccase.

## 1. Introduction

Concentrated emulsions are widely used in the cosmetics, personal care, oil recovery, and food industries to provide desired properties (smooth and soft texture, uniformity, stability, viscosity, moisture retention, and high carrying capacity) of particular products (e.g., creams, mayonnaise, and lotions) and to reduce storage and transportation costs [1]. In most cases, concentrated emulsions require large amounts of surfactants (5–50 vol % of the continuous phase), such as polysorbate-80 (Tween 80), which limits the application of concentrated emulsions [2,3]. The increasing customer demand for natural over synthetic ingredients and the rapid growth of functional foods requiring “green” additives represents an opportunity to use bioemulsifiers extracted from natural resources. The use of proteins, polysaccharides, and their mixtures as bioemulsifiers is becoming increasingly important due to their high versatility and environmental acceptability [4]. In addition, solid particles, such as polysaccharide, dextrin, modified starch, and fiber, also can be used as emulsifiers to stabilize emulsions (Pickering emulsions) [5,6,7,8]. However, few applications for Pickering emulsions have been found for commercial food products.

Coconut milk is an important vegetable protein beverage and is extremely popular worldwide [9]. In general, concentrated coconut milk (CCM) is initially produced and then diluted to yield the final product (coconut milk). It has been reported that coconut proteins act as emulsifiers to stabilize CCM [10], while, depending on the pH value of the emulsion, the proteins can be in different aggregation states and this can affect the stability. In addition, the high concentration of oil is another factor causing the instability of CCM. Previous researchers made some attempts to study the emulsification of coconut protein. Onsaard et al. found that coconut protein fractions can be used to prepare oil-in-water emulsions but cannot be used to create emulsions as stable as those formed by whey protein isolates [10]. Patil and Benjakul studied albumin and globulin fractions in coconut protein separately and discovered that the globulin fraction plays a more important role in emulsion stabilization than albumin [9]. Adding food gums is a simple and effective way to increase the stability of protein emulsions. Formation of complexes between proteins and polysaccharides can be achieved via electrostatic interactions, hydrogen bonds, hydrophobic interactions, and covalent interactions [11]. The coating of oil droplets with a protein–polysaccharide complex leads to steric repulsive forces that prevent the droplets from aggregating, thus thicker interfacial membranes consisting of multiple layers of biopolymers can remain stable for longer periods of time [12]. Furthermore, adding food gums can increase the viscosity of the continuous phase, hence retarding phase separation and gravity-induced creaming. Double-layered emulsions stabilized by protein–polysaccharide complexes (β-lactoglobulin–sugar beet pectin, sodium dodecyl sulfate (SDS)–chitosan and SDS–fish gelatin) showed more stability than those stabilized by corresponding proteins alone [13,14,15,16]. However, few studies have been conducted on the formation, especially via covalent binding, of coconut protein–polysaccharide complexes to improve the stability of CCM.

A relatively new type of pectin, sugar beet pectin (SBP), which is extracted from sugar beet pulp, was used as an emulsifier in our previous studies [17,18,19]. SBP differs from pectins obtained from other sources, and tends to have a higher degree of acetylation and a greater number of neutral sugar side chains as well as significantly larger numbers of feruloyl groups attached to the galactose and arabinose side chains [18]. Due to these unique structural characteristics, the existence of ferulic acid moieties esterified to the arabinose side chain at the backbone of rhamnogalacturonan I in SBP provides a pathway for enzyme-catalyzed oxidative crosslinking of SBP by oxidoreductase enzymes such as laccase [20]. The previously described process results in the irreversible formation of covalent crosslinks between beet pectin molecules. Covalent bonds are far stronger than electrostatic interactions, hydrogen bonds, or hydrophobic interactions. It is interesting to investigate the effect of oxidase on the emulsifying stability of emulsions stabilized by SBP and protein in a CCM model.

The purpose of the present study is to establish a model emulsion to simulate the CCM system. To investigate the effects of SBP on the stability of primary emulsions stabilized by coconut protein only, secondary emulsions were created by adding SBP to the primary emulsions. In addition, the emulsifying effect of SBP with different degrees of crosslinking was studied by adding different amounts of laccase to the secondary emulsion. The stability of the emulsions was evaluated by their interfacial tension, zeta potential, particle size distribution, and rheological properties, as well as by the assembly of SBP and coconut protein on the emulsion interface. Finally, the findings in the model were applied to a real CCM. The findings of this study are important for the application of laccase-treated SBP protein–stabilized CCM in the production of food and beverages.

## 2. Results and Discussion

### 2.1. Interfacial Tension and Charges

**Figure 1:**
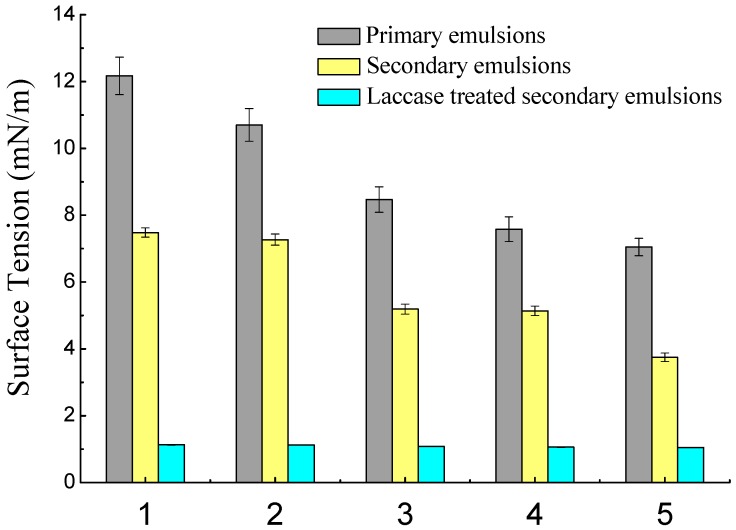
Interfacial tension of primary emulsions (1, 2, 3, 4, and 5 represent protein content of 0.8 g/100 g, 1.8 g/100 g, 2.8 g/100 g, 3.8 g/100 g, and 4.8 g/100 g, respectively); secondary emulsions (protein content was 3.8 g/100 g, and 1, 2, 3, 4, and 5 represent sugar beet pectin (SBP) content of 0.01 g/100 g, 0.02 g/100 g, 0.05 g/100 g, 0.10 g/100 g, and 0.15 g/100 g, respectively); laccase-treated secondary emulsions (protein content was 3.8 g/100 g, SBP content was 0.10 g/100 g, and 1, 2, 3, 4, and 5 represent laccase content of 2 U/100 g, 4 U/100 g, 6 U/100 g, 8 U/100 g, and 10 U/100 g, respectively).

This section describes the interfacial tension between coconut oil and a solution containing coconut protein, a solution containing coconut protein and SBP, and a solution containing laccase-catalyzed SBP and protein. As shown in Figure 1, the interfacial tension decreased as the protein content in the emulsion increased, which was due to both the hydrophilic and hydrophobic groups on the protein molecules. This shows that it is the coconut protein that acts as the emulsifier in coconut milk. Notably, when the protein concentration was 3.8 g/100 g, the same as the protein content in CCM, the interfacial tension was 7.58 mN/m. The interfacial tension decreased even when the coconut protein reached 4.8 g/100 g, which indicated that the surface of oil droplets was not completely covered by the protein and there was still room for more adsorption.

Similar to the primary emulsions, the interfacial tension of secondary emulsions decreased from 7.40 to 3.66 mN/m with the addition of SBP. The decrease of interfacial tension of the secondary emulsions might be attributable to the SBP, which also has good amphiphilic properties [21]. As mentioned above, the surface of oil droplets was not saturated, so the added SBP in the secondary emulsions may adsorb to oil droplets. Chen, Luo, and Fu reported that hydrophobic groups (ferulic acid and proteinaceous materials) in SBP could be adsorbed onto the surface of emulsion droplets, decreasing the interfacial tension between the water and oil phases. On the other hand, it is possible that soluble complexes are formed by the interactions between negatively charged polysaccharides and partially positively charged polypeptide regions on unfolded proteins [22]. After the addition of laccase, this decrease was pronounced. The maximum interfacial tension was less than 1.14 mN/m, and with increasing laccase, the interfacial tension decreased. Laccase induced conjugation in SBP through ferulic acid, and that conjugation was confirmed by measuring the reduced ferulic acids in laccase-treated SBP, resulting in an increase in the emulsifying capacity.

**Figure 2:**
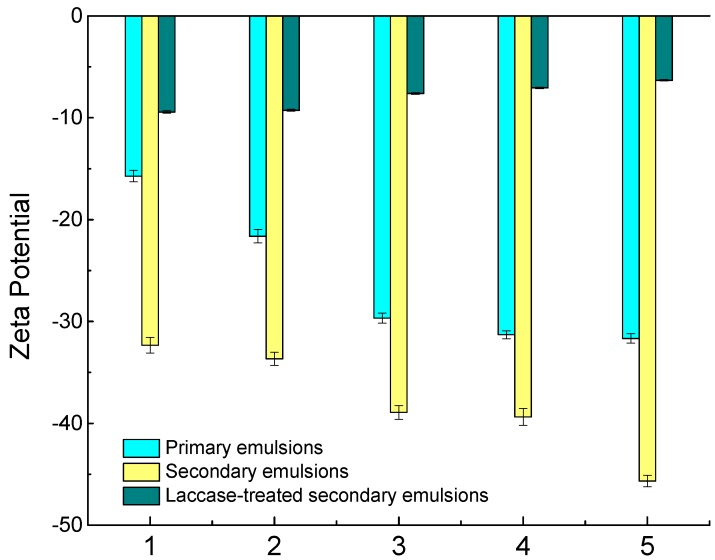
Zeta potential of primary emulsions (1, 2, 3, 4, and 5 represent protein content of 0.8 g/100 g, 1.8 g/100 g, 2.8 g/100 g, 3.8 g/100 g, and 4.8 g/100 g, respectively); secondary emulsions (protein content was 3.8 g/100 g, and 1, 2, 3, 4, and 5 represent SBP content of 0.01 g/100 g, 0.02 g/100 g, 0.05 g/100 g, 0.10 g/100 g, and 0.15 g/100 g, respectively); laccase-treated secondary emulsions (protein content was 3.8 g/100 g, SBP content was 0.10 g/100 g, and 1, 2, 3, 4, and 5 represent laccase content of 2 U/100 g, 4 U/100 g, 6 U/100 g, 8 U/100 g, and 10 U/100 g, respectively).

Zeta potential, a measure of mutual exclusion or attraction between dispersed particles, is an important indicator of the stability of colloid dispersions. Its value is closely related to the stability of the colloidal dispersion system. Coconut protein has a negative net charge at pH 6.8, which is above the isoelectric point (pH 4.0) [23]. As shown in Figure 2, the zeta potential of the primary emulsion was reduced from −16 mV to −32 mV with an increase of the protein content from 0.8 to 4.8 g/100 g. As the protein content of the emulsions increased, the amount of adsorbed protein on the oil droplets increased. The negative charge on the droplets reached a value of −32 mV when the protein concentration exceeded 3.8 g/100 g (the protein content in CCM). According to Onsaard et al., the surface charge of coconut milk (diluted CCM) was −26 mV, which is similar to the value found in this study [10].

The zeta potential of the secondary emulsion decreased from −32 to −46 mV when 0.10 g/100 g of SBP was added, which indicates a drastic decrease compared to the primary emulsion. A similar situation was discovered in a 0.05 wt % emulsion containing β-lactoglobulin and 0.02 wt % beet pectin at pH 7, which was much lower than the emulsion without SBP [24]. As mentioned above, both direct adsorption of SBP to the oil droplets via ferulic acid and the proteinaceous materials and the formation of SBP and coconut protein complexes are possible reasons for the reduction of zeta potential of the oil droplets. The relatively high negative charge of the oil droplets stabilized them against flocculation, which increased the electrostatic and steric repulsion between the droplets and reduced the van der Waals attraction.

Compared to the primary and secondary emulsions, the zeta potential of the emulsion was markedly less negative when laccase was added. The zeta potential increased from −9.5 mV to −6.0 mV as the laccase content increased. Similar to the findings of Jung and Wicker, the zeta potential of laccase-treated SBP was less negative than that of SBP, changing from −40.5 mV to −32.0 mV [22]. It was deduced that the presence of laccase might contribute to the burying of negative carboxyl groups within a more highly branched conjugated SBP. The charges of the laccase-treated secondary emulsions decreased, indicating that the electric interaction between the oil droplets became weaker, making it easier for them to coagulate in the laccase-treated secondary emulsions than in the primary and secondary samples.

### 2.2. Particle Size Distribution and Microscopic Structure

**Figure 3:**
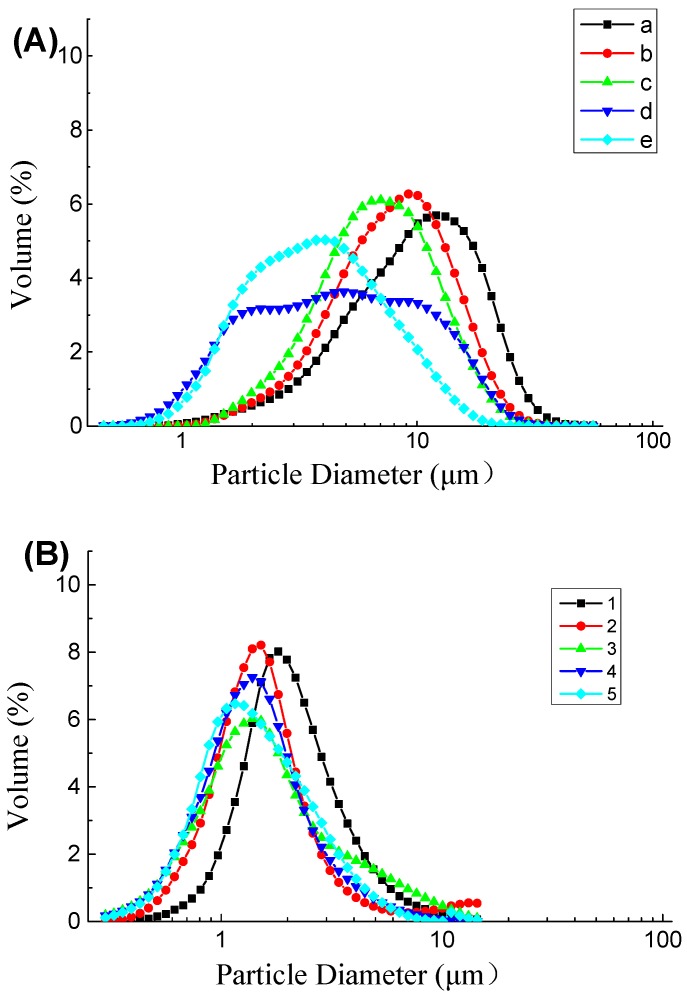

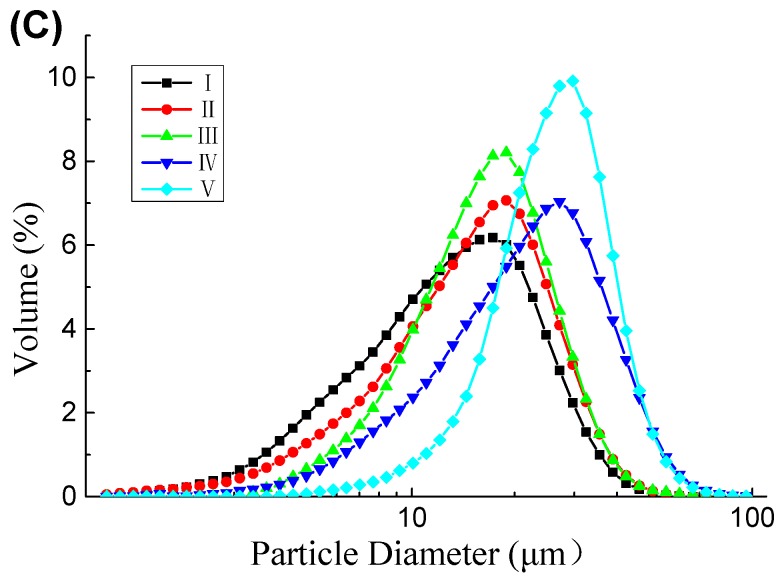
Particle size distribution of (**A**) primary emulsions (a, b, c, d, and e represent protein content of 0.8 g/100 g, 1.8 g/100 g, 2.8 g/100 g, 3.8 g/100 g, and 4.8 g/100 g, respectively); (**B**) secondary emulsions (1, 2, 3, 4, and 5 represent protein content of 3.8 g/100 g and SBP content of 0.01 g/100 g, 0.02 g/100 g, 0.05 g/100 g, 0.10 g/100 g, and 0.15 g/100 g, respectively); and (**C**) laccase-treated secondary emulsions (I, II, III, IV, and V represent protein content of 3.8 g/100 g, SBP content of 0.10 g/100 g, and laccase content of 2 U/100 g, 4 U/100 g, 6 U/100 g, 8 U/100 g, and 10 U/100 g, respectively).

**Figure 4:**
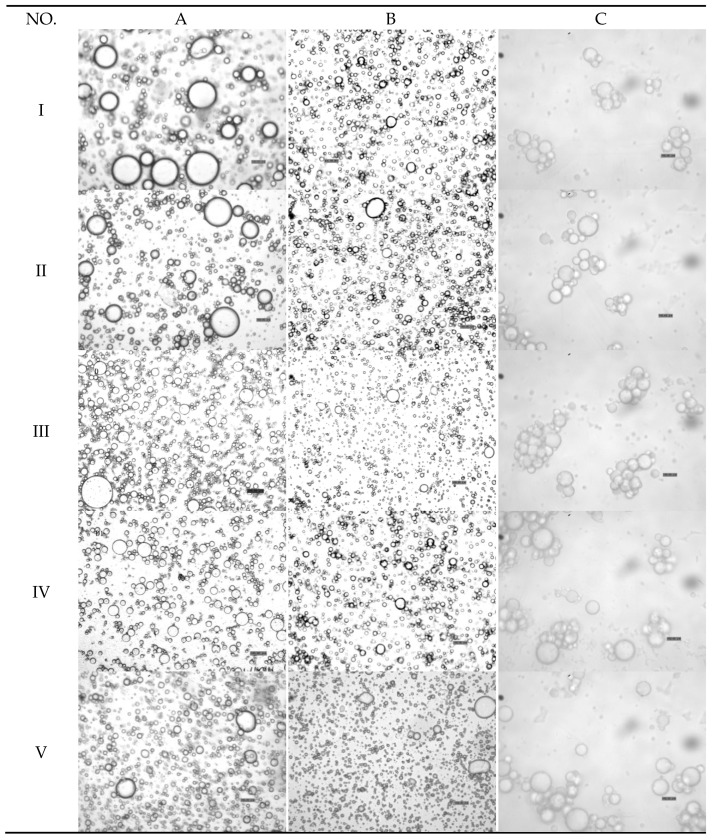
(**A**) Microphotographs of primary emulsions I, II, III, IV, and V represent protein content of 0.8 g/100 g, 1.8 g/100 g, 2.8 g/100 g, 3.8 g/100 g, and 4.8 g/100 g). (**B**) Secondary emulsions (protein content was 3.8 g/100 g; I, II, III, IV, and V represent SBP content of 0.01 g/100 g, 0.02 g/100 g, 0.05 g/100 g, 0.10 g/100 g, and 0.15 g/100 g, respectively). (**C**) Laccase-treated secondary emulsions (protein content was 3.8 g/100 g; SBP content was 0.10 g/100 g; I, II, III, IV, and V represent laccase content of 2 U/100 g, 4 U/100 g, 6 U/100 g, 8 U/100 g, and 10 U/100 g, respectively). Scale bar represents 10 μm.

As shown in Figure 3, the measured particle size distributions of most of the emulsions were unimodal, and the secondary emulsion exhibited the narrowest particle size distribution. In accordance with the tendency of interfacial tension and zeta potential, the D[4,3] values of the emulsions decreased as the protein content in the emulsions increased. The particle size of the primary emulsion was distributed from 0.40 to 60.00 μm, and the D[4,3] values of the primary emulsion decreased from 10.02 to 4.21 μm (Table 1). These results might be caused by the adsorption of coconut protein molecules to the surface of the droplets produced during homogenization, when they form a protective coating that prevents the droplets from aggregating, e.g., flocculating and/or coalescing. Additionally, the adsorbed proteins reduce the oil–water interfacial tension, thereby facilitating further disruption of lipid droplets during homogenization and leading to smaller droplet sizes. After homogenization, the coconut oil appeared in the form of oil droplets, as shown in Figure 4. Microphotographs of the primary emulsions directly reflected the sizes of the oil droplets in the emulsions, which corroborated the decreasing mean particle diameter.

The particle size in the secondary emulsion was distributed from almost 0.10 to 20.00 μm (Figure 3), which was smaller than that of the primary emulsion. The average particle size decreased from 2.77 to 1.60 μm as the SBP content in the emulsion increased (Table 1). In the secondary emulsions, protein was adsorbed at the oil–water interface, followed by stabilization promoted by pectin adsorption building a bilayer on the droplets formed through homogenization. The increases in the charges and thickness of the adsorbed polymer layer tended to stabilize the emulsion. Therefore, the adsorption of the pectin molecules to the droplet surfaces stabilized them against flocculation, which can be attributed to the increases in electrostatic and steric repulsion between the droplets and the reduction of van der Waals attraction.

As shown in Figure 3 and Figure 4, the particle diameter was distributed from 0 to 100 μm. The mean particle diameter increased from 13.88 to 26.17 μm, which was much larger than the particle sizes in the primary and secondary emulsions. The reason for this phenomenon may be that laccase crosslinked with the oil droplets formed clusters, which was confirmed in Figure 4 (column C). As mentioned above, the zeta potential was less negative in this condition than in the primary and secondary emulsions. Furthermore, this result can be attributed to the reduction of the electrostatic repulsion between the droplets (Figure 2).

### 2.3. Rheological Studies

**Figure 5:**
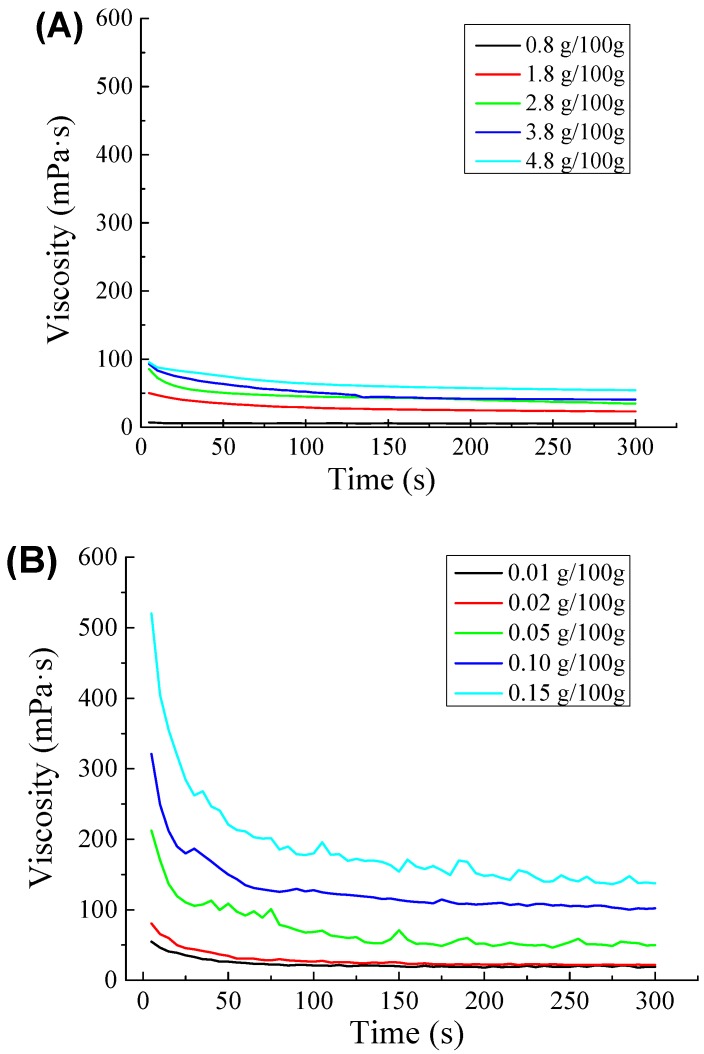

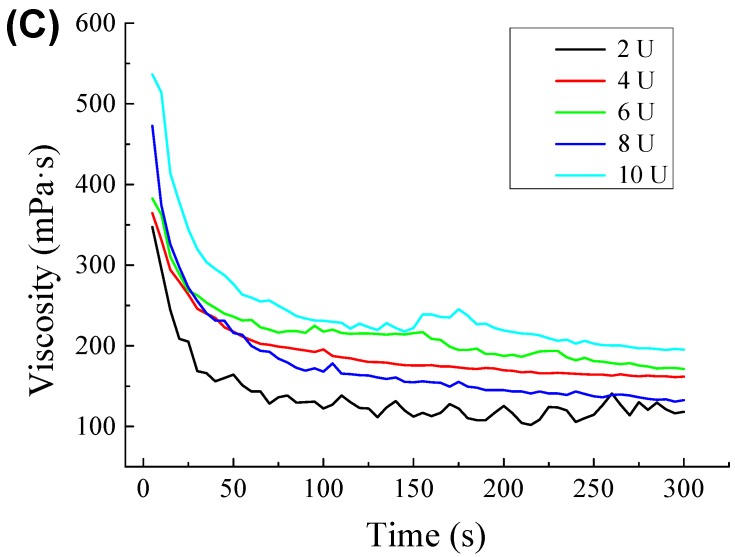
Change over time in viscosity of (**A**) primary emulsions (protein content was 0.8 g/100 g, 1.8 g/100 g, 2.8 g/100 g, 3.8 g/100 g, and 4.8 g/100 g); (**B**) secondary emulsions (protein content was 3.8 g/100 g, and SBP content was 0.01 g/100 g, 0.02 g/100 g, 0.05 g/100 g, 0.10 g/100 g, and 0.15 g/100 g); and (**C**) laccase-treated secondary emulsions (protein content was 3.8 g/100 g, SBP content was 0.05 g/100 g, and laccase content was 2 U/100 g, 4 U/100 g, 6 U/100 g, 8 U/100 g, and 10 U/100 g). Viscosity was tested when the temperature was 25 °C, and the speed was 20 rpm.

As shown in Figure 5, the apparent viscosity of all the emulsions decreased with shearing and showed the shear-thinning phenomenon. Obviously, the emulsions were all pseudoplastic fluids. As shown in Figure 5A, the apparent viscosity of the emulsions increased with increasing protein content. However, the emulsion stabilized with 0.8 g/100 g of protein showed almost no shear thinning. Most colloidal particles were entangled with each other when the emulsions were at rest. The relatively scattered chain-like particles rolled and rotated to shrink into clumps due to the shear stress between the flow layers, reducing the mutual hooking and decreasing the viscosity. For the same reason, the secondary emulsions showed obvious shear thinning (Figure 5B). Therefore, the viscosity of secondary emulsions was much higher than that of primary emulsions because of the thickening properties of SBP. Because the lower pectin content produced less viscous emulsions, the systems approached Newtonian behavior. The observed tendency toward Newtonian behavior reveals the emulsion stability against shear, which can be attributed to an adequate balance between the stabilizing biopolymers and the droplet size distribution, structure development, and lower resistance to flow. In Figure 5C, when compared to the other two groups, the laccase-treated secondary emulsions exhibited shear thinning and became more viscous. The change in viscosity might be associated with the gelation of SBP induced by the laccase-catalyzed crosslinking of ferulic acid groups, beyond which a network developed. The formation of a network structure gave rise to the increase in viscosity and was also responsible for the shear thinning when the formed network was destroyed [25].

### 2.4. Creaming Stability Measurements

**Figure 6:**
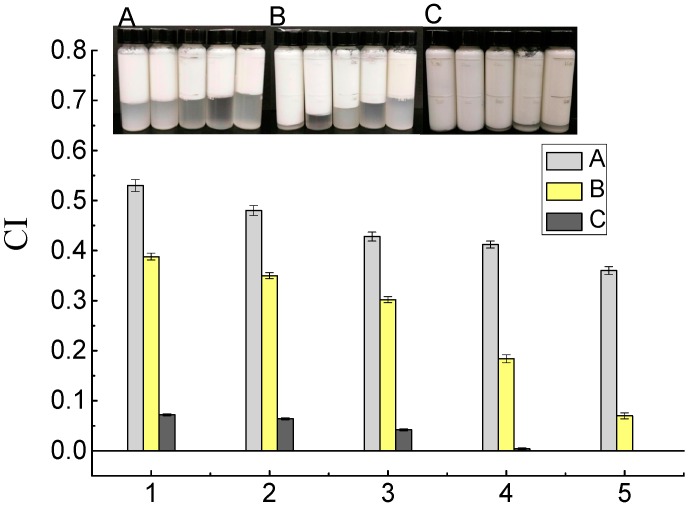
Creaming index (CI) of primary emulsions (1, 2, 3, 4, and 5 represent protein content of 0.8 g/100 g, 1.8 g/100 g, 2.8 g/100 g, 3.8 g/100 g, and 4.8 g/100 g, respectively); secondary emulsions (1, 2, 3, 4, and 5 represent protein content of 3.8 g/100 g; and SBP content was 0.01 g/100 g, 0.02 g/100 g, 0.05 g/100 g, 0.10 g/100 g, and 0.15 g/100 g); laccase-treated secondary emulsions (1, 2, 3, 4, and 5 represent protein content of 3.8 g/100 g, SBP content of 0.10 g/100 g, and laccase content of 2 U/100 g, 4 U/100 g, 6 U/100 g, 8 U/100 g, and 10 U/100 g).

**Figure 7:**
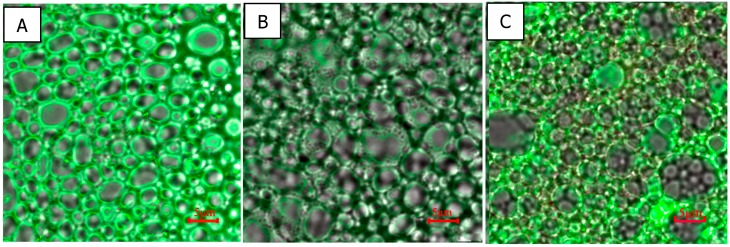
Laser confocal micrographics of (**A**), primary emulsion, (**B**), secondary emulsion, and (**C**) laccase-treated secondary emulsion.

The storage stability of the emulsions was also assessed by the creaming index (CI), and the results are shown in Figure 6. The primary emulsions creamed relatively extensively (CI > 0.35) and separated into layers after 24 h of storage. The CI of primary emulsions decreased from 0.53 to 0.36 as the protein content increased. The aggregation of oil droplets contributed to rapid creaming. In addition, the low viscosity accelerated the upward movement of the droplets [26]. Due to its surface-active properties and its ability to modify the rheological properties of colloid systems, SBP has been used as a stabilizer in emulsions. With the addition of SBP, the CI of the secondary emulsions was much lower (from 0.068 to 0.39). As shown in Figure 5, the secondary emulsions had higher viscosity, which increased the resistance to the accumulation of oil drops and floating of the cream. As mentioned above, some of the SBP was strongly adsorbed to the oil–water interface and some was attached to coconut protein. Both of these processes can stabilize emulsions for a longer period than coconut protein alone, probably by enhancing the electrostatic repulsion among the emulsified droplets. Littoz and McClements also found that emulsions containing lipid droplets coated by β-lactoglobulin and SBP had much better stability than those coated by β-lactoglobulin alone [24]. Laccase-catalyzed secondary emulsions have almost no layering, as shown in Figure 6A. It is possible that the entire emulsion network structure was formed and the droplets were fixed in cages, which blocked the upward movement of flocculated oil droplets (Figure 7C). In addition, a certain amount of SBP that was distributed in the continuous phase and was crosslinked resulted in an increase in viscosity. This process was also confirmed by Albano and Nicoletti (2018), who showed that oil droplets were coated by the protein and entrapped by the pectin, which formed a network in the continuous phase, stabilizing the system [27]. Perrechil and Cunha evaluated confocal laser micrographs of emulsions and found that the protein remained around the oil droplets, while the excess protein was homogeneously distributed in the continuous phase [28]. The fluorescence signals confirmed the presence of a protein layer adsorbed on the emulsion droplets; fluorescence was also detected when pectin was used as a second layer, indicating the presence of regions covered by large amounts of methoxyl pectin.

### 2.5. Verification Experiment and Mechanisms of Action of SBP and Laccase

To verify the method in the experiment and improve the stability of real CCM, SBP was added to coconut milk at 0.05 g/100 g. In addition, 10 U/100 mL laccase was added to secondary coconut milk. The CIs of the CCM, SBP–CCM, and laccase-treated SBP–CCM were 0.42, 0.32, and 0.01, respectively. Results showed that the CI of the coconut milk was similar to that in the emulsion model (Figure 6).

This suggests that the stability of real CCM can be improved by adding SBP and laccase, and the emulsion model (water/coconut oil/coconut protein) can be used to simulate CCM.

CCM is an unstable system and tends to break down during storage. The stability of emulsions requires the addition of emulsifiers and/or stabilizers. As shown in Figure 7A, coconut protein acts as an emulsifier because it is amphiphilic, reducing the interfacial tension at the oil–water interface and producing a film coating the oil droplets, preventing their aggregation to a certain degree. Polysaccharides such as SBP could act as stabilizers by increased gelation in the viscosity of the continuous aqueous phase. A complex of proteins and polysaccharides can be formed through electrostatic interactions, hydrogen bonds, hydrophobic interactions, and covalent interactions. In this research, as shown in Figure 7B, part of the negatively charged SBP might be electrostatically linked to positively charged moieties in the coconut protein and formed complexes. Confocal laser scanning microscopy (CLSM) showed that oil droplets were coated by the protein (in green) and covered by the pectin (in red), indicating that the protein was present at the interface and that pectin was (mainly) present in the continuous phase, stabilizing the system. The existence of FA moieties in SBP provided a way for the enzymatic catalysis of SBP to promote its oxidative crosslinking. Such covalently crosslinked structures were generally strong and stable. The crosslinked SBP formed a network in the continuous phase, and the oil droplets were entrapped in it. Therefore, the emulsions can avoid delamination and remain stable during measurements.

## 3. Materials and Methods

### 3.1. Materials

CCM was provided by the Taifengyuan Food Company (Haikou, China). SBP was obtained from CPKelco (San Diego, CA, USA). According to our previous reports (Chen, Fu, and Luo, 2015; 2016), the composition of SBP includes rhamnose (6.72 ± 0.03%), arabinose (9.03 ± 0.04%), galactose (9.86 ± 0.07%), glucose (0.66 ± 0.00%), xylose (1.00 ± 0.01%), galacturonic acid (43.57 ± 0.13%), protein (5.20 ± 0.02%), ferulic acid (1.21 ± 0.01%), and calcium (0.82 ± 0.03%) and has a degree of methylation (67 ± 0.4) and acetylation (23.9 ± 0.2) [17,19]. Laccase was purchased from the Aladdin Industrial Corporation (Shanghai, China). Milli-Q water was used in the solutions and emulsions and all chemicals were of analytical grade unless noted otherwise.

### 3.2. Determination of Coconut Protein and Fat in Coconut Milk

The protein content in the coconut milk was assayed by Kjeldahl determination [29]. The nitrogen content of CCM was analyzed by using a combustion method in which the protein content was calculated by using a conversion factor of 6.25. The fat content in CCM was determined by using the modified Mojonnier ether extraction method [30].

### 3.3. Preparation of Coconut Proteins and Coconut Oil

Coconut proteins and coconut oil were obtained by centrifuging CCM at 10,000 g for 10 min at room temperature. The coconut oil was obtained from the top layer. The skimmed coconut milk was used to isolate the coconut proteins by adjusting the pH to 3.9 (0.1 M HCl) [10]. The precipitate was then redispersed into distilled water (pH 7.0) and reprecipitated (pH 3.9). This process was repeated 3 times, and the precipitate was freeze-dried to obtain coconut protein.

### 3.4. Preparation of Model Oil-in-Water Emulsions

To simulate real CCM, similar constituents of oil and protein (30.32 wt % and 3.80 wt %, respectively) were used in certain model emulsion as a reference. Primary emulsions stabilized by proteins only were prepared by dispersing coconut protein powder into a mixture of phosphate buffer (5 mM, pH 6.8) and coconut oil (30.32% final content) to obtain stabilizer concentrations of 0.8 wt %, 1.8 wt %, 2.8 wt %, 3.8 wt %, and 4.8 wt %. Secondary emulsions were prepared by adding SBP (0.01 wt %, 0.02 wt %, 0.05 wt %, 0.1 wt %, and 0.15 wt %) to the primary emulsion (3.8 wt % protein). Then, the secondary emulsion (3.8 wt % protein and 0.1 wt % SBP) was treated with laccase (2, 4, 6, 8, and 10 U/100 g). The pH value was adjusted to 6.5 using HCl (0.1 M) or NaOH (0.1 M) solution. Sodium azide (0.02 wt %) was added as an antimicrobial agent. The mixtures were homogenized using a high-speed homogenizer (IKA T25 basic, Staufen, Germany) at 11,000 rpm for 3 min to form coarse emulsions, which were subsequently homogenized over 3 passes using an ultrahigh-pressure homogenizer (Nano DeBEE, South Easton, MA, USA) operated at 50 MPa.

### 3.5. Interfacial Tension Measurements

All solutions that were used as continuous phase in primary, secondary, and laccase-treated emulsions were prepared and kept in plastic bottles before each measurement. The oil–water interfacial tension was created by producing a pendant drop (bottom-to-top) of oil from a J-shaped syringe needle (needle diameter 1.5 mm) into a bath of each aqueous solution in a quartz cuvette (24 mm × 24 mm × 21 mm). The drop images were recorded with a CCD camera on a DropMeter (A-60, Haishumai Company, Ningbo, China) and the interfacial tension was calculated by fitting the drop profile with the numerical solution of the Young–Laplace equation [31]. Each measured point was an average from 3 replicates.

### 3.6. Particle Size Distribution Determination

The particle size distribution of the samples was measured on a Malvern Mastersizer 2000 (Zetasizer Nano-ZS, Malvern Instruments, Worcestershire, UK). To avoid multiple scattering effects during measurement, the experiments were carried out after the freshly made samples were diluted 1000-fold with deionized water (the degree of obscuration was approximately 15%). Values of 1.45 and 0.001 were used for the refractive index and absorption index of coconut oil, respectively, and values of 1.33 and 0 were used for the refractive index and absorption index of the water dispersant, respectively. The average size of the droplets in the emulsions was assessed in terms of the volume-weighted mean diameter D[4,3] [32].

### 3.7. Optical Microscopy

Optical micrographs of the emulsions were taken by using an optical microscope (Alphaphot-2, YS2-H, Nikon Corporation, Japan) equipped with a digital camera (SPOT Idea, Diagnostic Instruments Inc., Detroit, MI, USA). A drop of the emulsion was placed on a glass microscope slide and then covered with a glass coverslip. A microscope magnification of 100× (10× for the eyepiece, 10× for the objective) was employed [33].

### 3.8. Zeta Potential Measurements

The zeta potential of the emulsions was measured by a phase analysis light-scattering instrument (Nano ZS90, Malvern Instruments, Worcestershire, UK). Emulsions were diluted 1000-fold with deionized water to avoid multiple scattering effects. The diluted emulsions were mixed thoroughly and then injected into the measuring instrument, a standard 4-sided, 1 cm quartz cuvette. A parallel plate electrode was inserted, and the cuvette was placed in a temperature-controlled holder in which the temperature was kept at 25 °C at all times. The zeta potential of each sample was calculated from the average of 5 measurements on the diluted emulsion. The results are reported as mean and standard deviation (SD).

### 3.9. Rheological Properties

Viscosity was determined on a temperature-controlled Brookfield viscometer (Brookfield Engineering Lab, Middleboro, MA, USA), which consisted of a cylindrical spindle (25 mm diameter, 90 mm height) rotating inside a machined tube (28 mm internal diameter, 135 mm height). The samples were placed in the tube and allowed to equilibrate to 25 °C for 5 min prior to measurement. Data were collected every 5 s when the temperature was 25 °C, and the speed was 20 rpm [34].

### 3.10. Creaming Stability Measurement

Freshly made samples were transferred into sealed 10 mL tubes (10 mm internal diameter, 100 mm height) with minimal headspace to minimize evaporation and stored at 37 °C for 24 h. The extent of creaming was characterized by a creaming index (CI, %) according to Chen with some modifications and was calculated as follows [17]:

CI = (Height of the serum layer/Total height of the emulsion) × 100%(1)

### 3.11. Confocal Laser Scanning Microscopy (CLSM)

The distribution of emulsifiers (coconut protein and SBP) was analyzed by CLSM. The fluorescent dyes rhodamine B and fluorescein isothiocyanate were used to stain coconut protein and SBP, respectively. The observations were made on a confocal laser scanning microscope (TCS SP5, Leica, Wetzlar, Germany) with a 40× objective lens. The emission wavelength of fluorescein isothiocyanate was 523 nm. The emission wavelength of rhodamine B was 582 nm. The green and red fluorescence modes were used to produce excitation wavelengths of 488 and 543 nm, respectively. Images were obtained from the 2 channels and then superimposed [27].

### 3.12. Statistical Analysis

Each treatment was performed in triplicate. Significant differences among samples (*p* < 0.05) were assessed by using the statistics program SPSS 18.0 (SPSS Inc., Chicago, IL, USA). Data are presented as mean ± SD.

## 4. Conclusions

In this study, three model emulsions—primary emulsions stabilized by coconut proteins only, secondary emulsions stabilized by the conjugation of SBP and coconut protein, and laccase-treated secondary emulsions—were prepared to investigate the effects of different factors (coconut proteins, coconut proteins + SBP, laccase-treated emulsions) on the stability of model emulsions and applying the methods on real CCM. Result showed that the interfacial tension of the primary emulsion decreased with the increase of protein content, and the interfacial tension decreased to 7.40 and 1.14 mN/m with the addition of SBP (0.01%) and laccase (2 U/100 g), respectively. The total charges in the emulsion increased with the addition of protein and SBP and was concentration dependent, but reduced when laccase was added. The secondary emulsion exhibited the narrowest particle size distribution (0.1~20 μm); however, laccase-catalyzed secondary emulsions showed the best storage stability and no layering when the laccase content reached 10 U/100 g. CLSM revealed that protein was adsorbed on the oil–water interface and SBP distributed in the continuous phase could undergo oxidative crosslinking by laccase. This work shows that the stability of coconut milk can be improved by adding SBP to form layer-by-layer structures built by the hydrogen bonds between coconut proteins. By crosslinking the SBP and coconut proteins through the catalysis of laccase, the system becomes viscous and more stable. The effects of pH, ionic strength, and thermal treatment on the stability of secondary and laccase-treated secondary emulsions will be addressed in our next study.

## Figures and Tables

**Table 1:** D[4,3] of the primary, secondary, and laccase-treated secondary emulsions.

	Protein (g/100 g)					SBP (g/100 g)					Laccase (U)				
	0.8	1.8	2.8	3.8	4.8	0.01	0.02	0.05	0.1	0.2	2	4	6	8	10
D[4,3] (μm)	10.02	8.52	7.73	6.13	4.21	2.77	2.31	2.16	2.10	1.60	13.88	15.80	16.83	20.57	26.17
